# Effect of a Four-Week Vegan Diet on Performance, Training Efficiency and Blood Biochemical Indices in CrossFit-Trained Participants

**DOI:** 10.3390/nu14040894

**Published:** 2022-02-20

**Authors:** Krzysztof Durkalec-Michalski, Adrian Domagalski, Natalia Główka, Joanna Kamińska, Damian Szymczak, Tomasz Podgórski

**Affiliations:** 1 Department of Sports Dietetics, Poznań University of Physical Education, 61-871 Poznan, Poland; glowka@awf.poznan.pl; 2 Department of Physiology and Biochemistry, Faculty of Physical Education and Sport, Charles University, 162 52 Prague, Czech Republic; 3 Home of Body Training Studio, AFB Marcelin Sp. z o.o, 60-324 Poznan, Poland; domagalski.adrian1996@gmail.com (A.D.); szymczak-damian@wp.pl (D.S.); 4 Department of Physiology and Biochemistry, Poznań University of Physical Education, 61-871 Poznan, Poland; jkaminska@awf.poznan.pl (J.K.); podgorski@awf.poznan.pl (T.P.)

**Keywords:** vegetarianism, high-intensity functional training, sports nutrition, nutritional support, exercise performance, strength

## Abstract

This interventional study examined the effect of a four-week vegan diet (Veg_D_) during a four-week high-intensity functional training (HIFT) on performance, training results and blood biochemical indices in female (*n* = 12) and male (*n* = 8) moderate-trained CrossFit participants. The whole study group performed the maximum number of repetitions with a load of 70% one repetition-maximum (1RM) and a modified Fight Gone Bad (FGB_Mod_) test before and after a dietary intervention (the group was divided to follow a Veg_D_ or a traditional mixed diet (Mix_D_)) in a randomised and parallel design. Pre-exercise resting blood samples were also analysed. There was a significant improvement in the number of repetitions performed at a load corresponding to 70% of 1RM in the classic squat in the Mix_D_ group (*p* < 0.001), and in the classic deadlift in the Veg_D_ group (*p* = 0.014). Furthermore, there was a significant improvement in the results of the FGB_Mod_ performance test after a Mix_D_. Moreover, an improvement in some exercises in the modified FGB_Mod_ test (Wall Ball after the Veg_D_ and the Mix_D_, and rowing after the Mix_D_) was also observed. However, differences between the Mix_D_ and the Veg_D_ groups were not clinically relevant. In conclusion, the short-term study conducted here indicated that a Veg_D_ in HIFT training positively affects strength endurance in the classic deadlift but is unlikely to be more beneficial in improving performance than a Mix_D_.

## 1. Introduction

In recent years, the popularity of diets based on plant products around the world is growing. The increased interest in vegetarianism is related to the widespread awareness of the society regarding ecology and environmental protection, the risks of animal-borne diseases and the positive impact of plant-based diets on health [1,2,3]. Among the many forms of vegetarianism, a vegan diet (Veg_D_) is of great interest due to its rejection of consumption of any animal and of animal origin products and animal-derived ingredients. A Veg_D_ may be promoted for its alleged health benefits, such as lowering LDL concentration and blood pressure, reducing the risk of cardio-respiratory or heart diseases, type II diabetes or cancer [4]. Despite the well-documented benefits of vegetarian diets among the general population, there are only sparse studies investigating the effect of restricted plant diets on exercise performance in highly trained athletes [2,4]. Nevertheless, nowadays, there is a growing number of high-level vegan athletes, which suggests that this nutritional strategy may be more appealing for some individuals [4]. However, it has to be underlined that the main disadvantage of following a Veg_D_ is the predisposition to macronutrients (essential amino acids, omega-3 fatty acids), vitamins (vit. B_12_ and vit. D) and minerals (iron, zinc, calcium, iodine) deficiencies, which may negatively impact performance efficiency [4,5].

In addition to the beneficial effects of plant-based diets, vegetarian diets also induce other physiological effects that may offer benefits in terms of improving exercise capacity. These include the reduction of fat mass, the inflammation process and exercise-induced oxidative stress, as well as an improvement of glycogen availability through an increase in complex carbohydrate intake [6]. Furthermore, the aforementioned reduction of body fat mass is associated with increased submaximal and maximal aerobic capacity [7], which may be translated into increased endurance performance [8]. Moreover, the influence of diet on maximal oxygen uptake (VO_2max_), through the impact on body mass, is important not only for competitive athletes, but also for untrained and moderately trained recreational athletes [6]. One of the modifications of athlete’s body composition—the reduction of fat mass is negatively correlated with aerobic and anaerobic capacity. Moreover, the stimulation of increasing the contribution of fat-free mass is positively correlated to aerobic and anaerobic capacity [9,10].

Similar to a Veg_D_, high-intensity functional training (HIFT), including CrossFit—the most common HIFT program—is also gaining popularity in the sports environment. Over the past few years, HIFT has become an appealing new type of training that appears to provide similar benefits to more traditional training programs with less time commitment [11]. Although there are several studies substantiating the benefits of high-intensity interval training (HIIT), less is known about the effects of the relatively new training modality, known as HIFT, which emphasizes functional, multi-joint movements via both muscle-strengthening and aerobic exercises. Moreover, it can be modified to any fitness level and elicits greater muscle recruitment than traditional repetitive aerobic exercises [11]. Evidence suggests that HIFT may have a number of physiological benefits in healthy adults [12]. These include a higher satisfaction and a stronger feeling of belonging in participants taking part in the HIFT program and it can be more rewarding than traditional training programs with potential health benefits [13]. Moreover, it may improve aerobic and anaerobic capacity as well as muscle strength and endurance in untrained women [14,15]. HIFT can also significantly improve body composition in medium-trained adult men and women by reducing body fat percentage and increasing bone mineral content [16].

There is scientific-based support for the possible beneficial effects of the use of a Veg_D_ or HIFT training on the improvement of sport (e.g., an increase in maximum strength and physical performance), health (e.g., normalization of glucose, cholesterol, HDL and LDL concentrations) and nutritional status (e.g., a reduction in the percentage of body fat, higher levels and availability of certain nutrients) indicators of athletes [6,11]. According to these remarks, in this study, we hypothesised that a Veg_D_ combined with HIFT would not be more effective than customary Mix_D_ in improving strength capabilities, exercise performance and blood biochemical indices of moderate-trained participants. Therefore, this study aimed to compare the effect of a four-week Veg_D_ vs. Mix_D_ during four weeks of controlled HIFT on exercise strength capacity, HIFT performance results and selected blood biochemical indicators in moderately trained CrossFit practitioners.

## 2. Materials and Methods

### 2.1. Participants

Twenty participants were enrolled in this study. All of them (12 females and 8 males) completed the study protocol and were included in the analyses (Table 1). All athletes participated in three monitored HIFT training sessions per week for four weeks. All of the participants regularly attended the same gym (Home of Body training studio in Poznan, Poland) and carried out HIFT for a period of approximately 12 months. Moreover, none of the participants followed a vegan/vegetarian diet prior to the study commencement. All participants followed a mixed diet, including products of animal origin. Participants were included in the study protocol based on several inclusion criteria. They were free from any physical constraints that might have affected the study results. Study participants had a valid and up-to-date medical certificate of the athlete’s ability to practice sports. In addition, they received health and activity questionnaires, in which they confirmed that there were no contraindications to physical activity, as well as any musculoskeletal, cardiovascular, respiratory and metabolic diseases that would limit their ability to exercise. Planning in the near future or being already pregnant women were also not enrolled in the study. Moreover, the participants of the study stated that they did not use any stimulants or take any medications, including supplements that may improve performance or anabolic androgenic steroids. All participants were asked to refrain from using dietary supplements during the study (including creatine, beta-alanine, caffeine, amino acids, “pre-workout boosters”, vitamins and minerals). People using drinks containing caffeine in the form of coffee or tea were able to still use it, apart from pre-workout nutrition. Potential participants who did not declare diet adherence and compliance with the study protocol throughout the duration of the study were excluded from the recruitment. 

The study protocol was reviewed and approved by the local institutional review board (Bioethics Committee at Poznan University of Medical Sciences, Poland, reference number: 389/20). Written informed consent was obtained from all participants before the study began. The document with the information on the study procedures for the participant also explained all the intervention procedures and presented the potential risks and possible benefits. All procedures were conducted in accordance with the ethical standards of the 1964 Declaration of Helsinki. The main studies were conducted from September to October 2020.

### 2.2. Study Design

The study protocol consisted of the implementation of a Veg_D_ and a Mix_D_ during the monitored HIFT training period in a randomised and parallel design. The primary outcomes were changes in HIFT-specific exercise performance and endurance strength. Secondary outcomes were the changes in selected blood biochemical markers after nutritional interventions.

#### 2.2.1. Study Protocol and Visits

**Figure 1:**
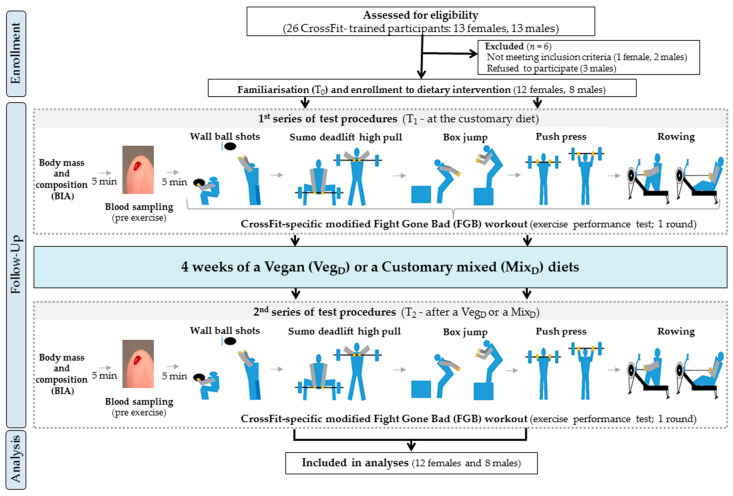
A flow chart of the study design.

The research protocol included three study visits (familiarisation with the entire protocol (T_0_), pre-treatment (T_1_) and post-treatment (T_2_) analyses; Figure 1). After T_0_, the participants were firstly enrolled by the authors and then randomly assigned (stratified randomisation based on HIFT exercise performance results) to the Veg_D_ or the Mix_D_ groups by an impartial biostatistician. To maintain the full specificity of the measurement conditions, the exercise tests were carried out: (a) in a sports club where the participants trained as usual on a daily basis (at the Home of Body training studio in Poznan, Poland), (b) at the same time of the day, in the morning hours, and (c) under constant temperature conditions (20–22 °C), three hours after consuming a standardised meal (carbohydrates: 2.0 g·kg^−1^, proteins: 20 g), as described previously [17,18]. Furthermore, despite the inclusion of the T_0_ period for additional familiarisation, the cross-training participants were already familiar with the CrossFit-specific workouts and exercise tests, as well as the entire study protocol, such as HIFT and strength training and dietary and physical activity questionnaire recording methods.

#### 2.2.2. Dietary Intervention

Before the implementation of the 4-week dietary intervention, a short individual course was conducted for the participants in the field of Veg_D_ or Mix_D_ recommendations. Both the Veg_D_ and the Mix_D_ were individualised to ensure weight maintenance throughout the dietary intervention, and diets were energetically normalised. Each participant received a monthly menu via e-mail with recommendations for a Veg_D_ or a Mix_D_ (including animal-derived products), which they started following the day after T_1_. After the dietary intervention, the second series of test procedures (T_2_) was conducted. Individual total daily energy requirements were estimated in accordance with the previously described methodology [17,19], also taking into account training sessions and activity during the day. Based on estimated energy expenditure and relevant recommendations [20] the final distribution of macronutrients in grams (carbohydrates: 4.5–5.5 g·kg^−1^·day^−^^1^, proteins: 1.5–2.0 g·kg^−1^·day^−1^, fats: 0.8–1.5 g·kg^−1^·day^−1^) was adapted. Study participants were asked to record their real daily diets, based on the diet recommendations received, using the MyFitnessPal mobile app (Under Armor Inc., Baltimore, MA, USA). This program contains a large and detailed food database and has been validated against paper-based food composition tables [21]. Furthermore, the diet adherence and study protocol compliance were supported and confirmed by personal contact of the qualified sports dietitian with each participant at least three times a week during their HIFT training and additional free-access contact via the Internet. Final diet intake is presented in Table 2 and Supplementary Material Table S1.

#### 2.2.3. A Four-Week High-Intensity Functional Training Protocol

The four-week training program, based on the principles of HIFT, was individually monitored. During the entire intervention, each HIFT training was conducted in the Home of Body training studio by a certified trainer in order to control the exercise technique, adhere to the programmed intensity and volume of the training and maintain all safety considerations. The workout of the day (WOD) scheduled for a specific day was the same training for each participant in the study. All training sessions consisted of a strength session followed by an aerobic training session. In the strength part of the training, progressive muscle load was used with the use of intensity from 60% to 100% 1RM for given exercises. The typical Olympic weightlifting exercises, strength movements with participants’ own body weight (e.g., squats, push-ups, pull-ups), non-traditional training methods (e.g., kettlebell swing, medicine ball throws) and other anaerobic exercises (e.g., skipping rope, rowing ergometer, sprint) were introduced to the training procedure. Moreover, in order for all subjects to have the opportunity to participate and complete the efforts, the supervising instructor introduced a regression of the exercises’ nature if the person was unable to perform a more complex task (e.g., classic push-ups, pull-ups, box jumps) or a more intense task. The regression exercises were selected in such a way that the same muscle parts were involved in the work as in the case of the main exercise, while reducing the intensity or difficulty of a given exercise (e.g., classic push-ups/women’s push-ups, pull-ups/pull-ups with the use of a rubber, box jumps/step jumps). If the participants were not able to complete the training on the specified day and at the appointed time, the participants could perform the training unit in the gym at a time more convenient for them, after agreeing with the instructor. In order to collect all the necessary data, a separate folder was prepared for each participant in a Microsoft Excel 2019 sheet (Microsoft Corporation, Redmond, WA, USA), in which the researcher recorded all the participants’ progress, and after the end of the four-week intervention, a collective analysis of all the results was performed. The four-week cycle consisted of two phases: (a) the initial preparation to undertake proper physical activity (medium-intensity training using own body weight, training units A and B)—1 week, and (b) the phase of a proper training with increasing intensity over time (training with additional load and greater intensity, training units C and D)—3 weeks (a detailed description of the particular exercises included in a certain phase and training unit can be found in the Supplementary Material Table S2).

### 2.3. Exercise Tests

#### 2.3.1. One Repetition-Maximum (1RM)

At T_0_, and in the period of two days before T_1_ and T_2_, participants were subjected to a preliminary study to determine their individual one repetition-maximum (1RM), which was necessary for individual adjustment of the load of 70% 1RM, vital to conduct a strength endurance test, a classic barbell squat and a classic deadlift. The study was carried out under the supervision of a certified personal trainer to ensure the appropriate technique of the exercises and belaying. Immediately prior to the 1RM assessment, all participants performed an identical warm-up consisting of an 8 min treadmill jog (6.5 km·h^−1^, 3.0 ascent), followed by 20 squats with own body weight, 20 lunges with own body weight and dynamic stretches of quadriceps muscles of the thigh marching, 10 repetitions for each limb, and stretches of biceps muscles of the thigh marching, 20 repetitions. The purpose of the warm-up was to prepare the muscles and joints for heavy effort, while minimizing fatigue during it, so as not to reduce the size of the weight lifted in the maximum repetition.

In the main part of the test, each participant performed, in accordance with the recommended procedure [22], two warm-up series of both squats and deadlifts with a weight of approximately 40–60% and 60–80% of their perceived maximum, respectively, in the amount of 8 and 3 repetitions, respectively. After performing a set with approximately 80% of the perceived maximum weight, single repetitions were performed, and the load was increased by 5 kg until the participant was unsure whether to perform the next repetition. Then, the weight on the bar increased by 2.5 kg.

For each exercise, 4–5 attempts were made to determine 1RM. A minimum rest of 3 min was allowed between each trial. Any 1RM attempt visually falling outside the range of motion criteria for both exercises, or in failure to observe technique, was considered a failure and was rejected. 

#### 2.3.2. Endurance Strength Evaluation

After at least two days of rest, a test was carried out to perform the maximum number of repetitions in the classic barbell squat and the classic deadlift with a load of 70% 1RM. The value of 70% 1RM was selected individually for each participant on the basis of the previously established maximum load in both exercises. Only the repetitions made in compliance with the movement criteria for both exercises and the appropriate technique were included. Immediately before the test, all participants performed the same warm-up sessions as described above (see Section 2.3.1). 

#### 2.3.3. Monitoring of a Specific HIFT Performance

Before (T_1_) and immediately after (T_2_) the four-week nutritional intervention, participants completed a modified HIFT-specific Fight Gone Bad test (FGB_Mod_). Due to the exercise arrangement scheme in this training, it was possible to compare the achievements before the study with the effects of the nutritional interventions. Immediately before the start of the FGB_Mod_, a certified trainer performed a warm-up consisting of an 8 min treadmill run (speed 8.0 km·h^−1^, climb 3.0), then 20 squats with own body weight, 10 repetitions of ‘fall get up’, 10 jumps on the chest and a dynamic stretching of large muscle parts.

The FGB_Mod_ required the participants to perform the following actions as quickly as possible, in the following order: (1) ‘Wall Ball’—squats with a medicine ball (9 kg for men, 6 kg for women) and throwing this ball up to a target (3.0 m for males, 2.75 m for females), (2) ‘Sumo Deadlift High Pull’—lifting the bar from the ground—‘sumo deadlift’ (60 kg for men, 40 kg for women), (3) ‘Box Jump’ (on the chest for men, on the step for women), (4) ‘Push Press’—pressing the bar over the head while standing (30 kg for men, 15 kg for women) and (5) rowing on an ergometer [18,23,24]. In each exercise, it was necessary to perform as many technically correct repetitions as possible within 1 min. On a ‘substitution’ call, the competitor had to move to the next station immediately to get the best result. One point was awarded for each repetition, with the exception of the rowing ergometer where the participant received one point for each ‘calorie’ expended. Each person had a single five-minute round to complete. The total FGB_Mod_ completion time and the number of points earned by each participant were recorded and used for analysis. All FGB_Mod_ tests were recorded using video recordings.

Furthermore, we innovatively implemented the modified FGB_Mod_ protocol consisting of: (1) carrying out one series of five exercises, (2) the barbell weight used during the ‘Sumo Deadlift High Pull’ exercise has been increased and that used during the ‘Push Press’ exercise has been reduced, respectively, and (3) within each exercise, the actual muscle work lasted one minute, and the total FGB_Mod_ completion time was also counted, taking into account the exercises and the transitions between stations and any breaks during the effort. All of these elements were used on purpose, in order to maximize the intensity of the HIFT-specific effort.

### 2.4. Blood Samples’ Analysis

Resting (pre-exercise) blood samples were collected from a fingertip of the nondominant hand using a disposable lancet-spike Medlance^®^ Red (HTL-STREFA, Łódź, Poland) with a 1.5 mm blade and a 2.0 mm penetration depth. The capillary blood was collected into: (a) a Microvette^®^ CB 300 tube (about 300 µL of capillary blood; Sarstedt, Nümbrect, Germany) containing EDTA dipotassium salt as an anticoagulant for haematological measurement using a 20-parametric automated Mythic^®^ 18 haematology analyser (Orphée, Geneva, Switzerland), and (b) a Microvette^®^ CB 300 Z tube (about 300 µL of capillary blood; Sarstedt, Nümbrect, Germany) with a clotting activator, in which the total cholesterol (cat. no. 7-204), high-density lipoprotein (HDL; cat. no. 7-279), low-density lipoprotein (LDL; cat. no. 7-280), triglycerides (cat. no. 7-253), iron (cat. no. 7-258), transferrin (cat. no. 7-210), ferritin (cat. no. 7-230), total protein (cat. no. 7-236), albumin (cat. no. 7-238), glucose (cat. no. 7-201), urea (cat no. 2-206) and creatinine (cat. no. 7-277) concentrations, as well as alanine aminotransferase (ALT; EC 2.6.1.2; cat no. 1-221) and aspartate aminotransferase (AST; EC 2.6.1.1; cat no. 1-222) activities were analysed. All the reagents used to determine the above-mentioned biochemical indices were from the PZ Cormay S.A. company (Łomianki, Poland) and measurements were performed using the Accent 220S automatic biochemical analyser (Cormay, Łomianki, Poland). Moreover, all markers related to the number of cellular components, such as leucocyte (WBC), granulocyte (GRA), lymphocyte (LYM), monocyte (MON), erythrocyte (RBC) and platelet (PLT) counts, as well as haemoglobin (HGB) and biochemical parameters, were calculated by taking into account the haematocrit conversion factor as it was previously described [17,25]. This approach was applied to avoid misinterpretation of blood parameters’ results due to different blood hydration statuses at the two study visits.

### 2.5. Statistical Analysis

Data are presented as mean values, standard deviation ($\bar{X}\pm \mathrm{SD}$) and the confidence interval (95% CI). The studied variables were checked for normality of distribution using the Shapiro–Wilk test. In order to compare the habitual and test diets (Veg_D_, Mix_D_), the FGB_Mod_ test results and blood haematological and biochemical markers before and after the nutritional interventions and the results of the repetitive training (A1 vs. A2), the paired Student’s *t*-test for indicators with normal distribution and the Wilcoxon rank-sum test for indicators with no normal distribution were performed. In order to compare the differences between the used diets (Veg_D_ vs. Mix_D_) for all tested parameters, the Student’s *t*-test for independent samples for indicators with normal distribution and the Mann–Whitney U test for indicators with no normal distribution were carried out. In order to compare the results of repetitive training (C1 vs. C2 vs. C3 vs. C4 vs. C5 vs. C6; D1 vs. D2 vs. D3), the ANOVA with repeated measures for indicators with normal distribution and the Friedman ANOVA test for parameters with no normal distribution were applied. The sample size met the study assumptions, as previously described [17,18]. The level of significance was set at *p* < 0.05. Statistical analysis was performed using the computer statistical package STATISTICA 13.3 (StatSoft, Inc., Tulsa, OK, USA).

## 3. Results

### 3.1. Assessment of Strength Endurance and Discipline-Specific Performance

**Figure 2:**
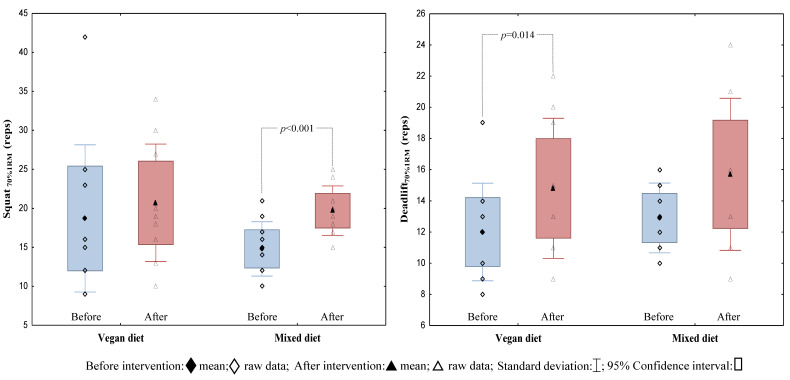
The results of the endurance strength in the classic squat and the deadlift before and after the 4-week nutritional intervention in the Veg_D_ and the Mix_D_ groups.

**Figure 3:**
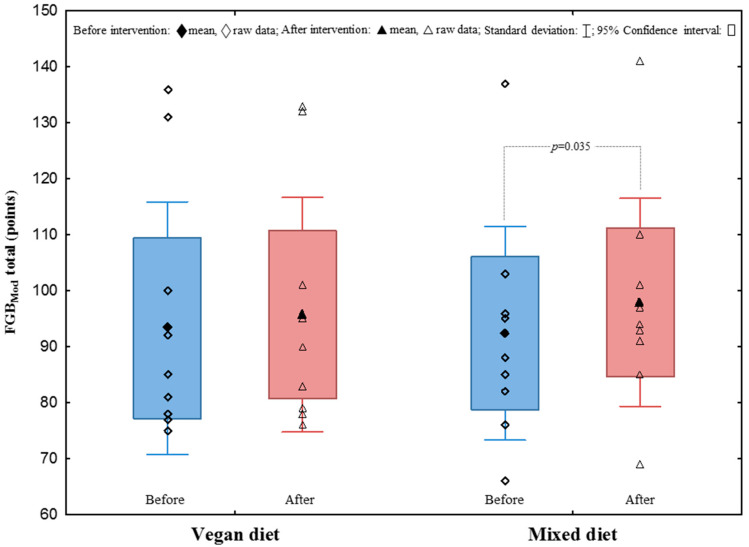
Total score in the FGB_Mod_ test.

After the study protocol, the average number of correctly performed classic deadlift and squat repetitions with an individualised load of 70% 1RM increased in the Veg_D_ and the Mix_D_ groups, respectively (Figure 2). Moreover, there was a significant improvement in the results of the FGB_Mod_ performance test after a Mix_D_ (Figure 3). Furthermore, an improvement in some exercises in the modified FGB_Mod_ test (Wall Ball after the Veg_D_ and the Mix_D_, and rowing after the Mix_D_) was also observed (Table 3). Nevertheless, comparing the two treatment groups, no significant differences of strength endurance and discipline-specific performance were found. 

### 3.2. The Influence of Diets on the Results of the Implemented Four-Week HIFT Intervention

The first phase of the four-week HIFT intervention, which was the initial preparation for proper exercise, consisted of two ‘A’ training units and a single ‘B’ training unit (Supplementary Material Table S2). No significant differences were observed in the mean number of series of exercises or in the average time of completing particular training units (A1, A2, B) in any of the interventional groups (Supplementary Material Table S3). Moreover, the evaluation of the results between the diets used (Veg_D_ vs. Mix_D_) did not show a statistically significant difference.

The second phase of the four-week HIFT intervention, which was the actual training phase of increasing intensity over time, consisted of six ‘C’ and three ‘D’ training units (Supplementary Material Table S3). In the Veg_D_ group, comparing C1 and C6 training sessions, there was a 12.4% increase in the average number of exercise series performed during the training unit. Changes in the average number of series of exercises performed between training C1 and C5, C1 and C6, and C2 and C6 were statistically significant. Comparing D1 and D3 training sessions, there was a 15.7% decrease in the average duration training unit when performing the same number of exercises with a given load. Differences in the mean duration of training D1 and D2, D1 and D3 as well as D2 and D3 were statistically significant (Supplementary Material Table S3).

Moreover, in the Mix_D_ group, comparing C1 and C6 training sessions, there was a 17.4% increase in the average number of exercise series performed during the training unit. Changes in the average number of series of exercises performed between training C1 and C6, and C2 and C6 were statistically significant. Differences in the average duration of training D1 and D3, and D2 and D3 were also statistically significant. In contrast, changes in the average D1 and D2 training durations were insignificant (Supplementary Material Table S3).

### 3.3. Evaluation of Haematological and Biochemical Indices

#### 3.3.1. Blood Haematological Markers

Blood haematological markers’ levels before and after the Veg_D_ and Mix_D_ nutritional interventions showed no statistically significant differences (Table 4). However, surprisingly, between-group comparisons revealed higher RBC counts (*p* < 0.047) after a Veg_D_ in relation to a Mix_D_.

#### 3.3.2. Lipid Profile Markers

Total, HDL and LDL cholesterol concentrations before and after the study showed no statistically significant differences between and within the Veg_D_ and the Mix_D_ groups (except the triglycerides concentration in the Mix_D_ group (*p* < 0.005) after nutritional intervention) (Table 5).

#### 3.3.3. Other Selected Biochemical Markers

There were no statistically significant changes in concentrations of other biochemical markers in both groups or between the groups before and after the study (results are combined in Table 6).

## 4. Discussion

The main purpose of this study was to investigate and compare the effect of a Veg_D_ and a Mix_D_ combined with a four-week HIFT on exercise capabilities in endurance strength at 70% 1RM, HIFT-specific performance and HIFT-training efficiency outcomes in CrossFit-trained practitioners. Additionally, the researchers sought to further examine selected haematological and biochemical blood markers’ changes, as a result of a diet modification. The main findings did not reveal crucial effects of the implemented Veg_D_ intervention on performance-, training- and biochemical-related outcomes.

Knowledge and capability to plan and compose a diet, especially with major product restrictions, make a vital impact on athletes’ health [5]. Athletes in general have higher requirements for energy, micro- and macro-nutrients, which is related mainly to the energy requirements of exhausting trainings [20]. Therefore, restricted diets, such as the vegan diet, may be considered deficient and potentially harmful for athletes’ exercise capabilities. The NURMI (Nutrition and Running High Mileage) Study, a cross-sectional design, revealed a higher prevalence of use of vitamin supplements’ intake in vegan endurance runners compared to vegetarian and omnivorous runner. Moreover, higher CHO and lower protein and fat intakes were observed in vegans compared to vegetarians and omnivores [26].

Within-group improvements in endurance strength (the number of repetitions performed with a load of 70% 1RM) in a classic squat in the Mix_D_ group (*p* = 0.001), and in a classic deadlift in the Veg_D_ group, were noted. Nevertheless, when comparing the results of both groups in these exercises, they were not statistically significant (for a classic squat and for a classic deadlift: *p* = 0.703 and *p* = 0.673, respectively). In support of these data, there are other studies showing strength improvements induced by HIFT [16,27], but no differences in 1RM improvements between the groups receiving animal and plant protein [27,28,29,30]. The results obtained by both groups after our four-week nutritional intervention during HIFT in a classic squat and a deadlift with a load of 70% 1RM may result more from the predisposition of people to a given exercise, and less from the type of nutrition model used. It turned out that some participants were much better at progressing in breaststroke repetitions than in breaststroke deadlifts, and *vice versa*. This idea is supported by the fact that participants performed the same HIFT protocol during the entire course of the study.

Furthermore, we found no improvements within the groups, nor differences between the groups in the first phase of the four-week HIFT intervention. After the four-week nutritional interventions, individual groups experienced a significant increase in the number of repetitions performed (C1–C6 training) and a significant improvement in the speed and a shorter time of completing training units (training D1–D3). Although some improvements (not significant) in some tasks of the HIFT-specific FGB_Mod_ performance were found in both groups, there were no changes between the groups after the intervention period. It is important to note that in contrast to previous studies [24,31], an innovative modified version of FGB was used, consisting of only one five-minute round of exercises. In previous work, it was shown that the classic 3-round FGB (15 min of effort with 2 min of break) is strongly related to aerobic fitness and capacity [24]. However, in the current study, it was intended to maintain the highest exercise intensity that would be conducted in mainly anaerobic conditions; therefore, it was decided to implement one FGB round, as indicated above. Therefore, it was hypothesised that it does not seem that the increased supply of carbohydrates characteristic of plant-based diets has a positive effect on the performance in short and intense HIFT/cross-training exercises. There are limited studies concerning plant diet or plant protein sources in terms of HIFT improvements. Similar results were observed in the study by Banaszek et al. [27], where no significant improvements were found for WOD1 or WOD2 as a result of eight weeks of HIFT with no differences found between whey and pea protein conditions. However, it is in contradiction to improvements in WOD found by Feito et al. [16] after 16 weeks of HIFT and by Outlaw et al. [32] and Kephart et al. [33] after 6 and 12 weeks of CrossFit training, respectively.

The current study interestingly showed no differences in blood biochemical indices after diet interventions between the groups, except significantly higher values of red blood cells in the Veg_D_ group, which probably occurred due to higher values of this marker at the beginning of the study protocol. For this reason and due to its limited clinical importance, this observation should be approached with caution. In addition, basal indices were not carefully investigated in previous studies. In a systematic review and meta-analysis from 2019, Craddock et al. [34] showed that two out of four studies reported a lowered total lymphocyte count in vegetarian-based groups, however in both studies lymphocyte counts were within normal reference ranges. Monocytes count was examined in three studies and showed non-significant results. Platelets remained stable in both study types. A further study by Lederer et al. [35] showed no differences between the groups after nutritional interventions (Veg_D_ vs. meat-rich diet) in haemoglobin concentration and lymphocytes count, but total leukocyte and platelets counts were significantly lower after a Veg_D_ compared to a meat-rich diet (but still remained within the reference range in both). Moreover, red blood cell counts did not significantly change.

It should be mentioned that endurance training tends to reduce iron stores, which is especially highlighted when iron needs are not adequately met in the diet [36]. Widespread concerns for iron deficiency in vegetarian diets observed in athletes, supported by studies reporting a high prevalence of iron depletion and anaemia, especially in females [37,38], are based on the poor bioavailability of iron from plant sources [36]. On the other hand, iron deficiency anaemia rarely occurs in vegetarian athletes and a mild iron deficiency is not likely to impair performance [36]. Surprisingly, contrary to what is commonly believed, this study did not reveal any significant changes in iron metabolism markers (iron, transferrin, ferritin) between the groups after the Veg_D_ and Mix_D_ interventions. On the contrary, Shaw et al. [39] examined iron intake and status in vegetarian and non-vegetarian students and showed lower ferritin and higher transferrin concentrations in vegetarians, which indicated lower iron stores and higher organism’s demand for this microelement. Moreover, Snyder et al. [40] presented lower iron stores and lower serum ferritin concentration in semi-vegetarian athletes than in runners not restricting red meat consumption, but no differences in serum iron, haemoglobin concentration and haematocrit value. Nebl et al. [41] showed significantly higher ferritin concentration in omnivorous than lacto-ovo-vegetarians and vegan athletes (but no differences among women) and depleted iron stores in all groups. These above-mentioned studies, however, included mainly endurance athletes, and we investigated people performing HIFT, and hence the different nature of the effort could have influenced the results observed in this regard. Moreover, the frame of time used in the current study may have been too short. Although, to our knowledge, there are no unambiguous studies in humans indicating how quickly blood indices’ levels should change in response to diet and the data so far only include studies on rats [42,43].

Furthermore, a vegetarian diet, which is low in saturated fats and may be high in polyunsaturated and omega-6 fatty acids, may play a beneficial role in reducing cholesterol and triglycerides concentrations. As a consequence, it can be beneficial to the health status as following a Veg_D_ may reduce the risk of developing potentially fatal diseases, such as cardiovascular diseases [44]. We did not observe significant differences in lipid profile markers between the Veg_D_ and the Mix_D_ groups. The lack of statistically significant differences for total cholesterol and HDL and LDL cholesterol fractions in participants following a Veg_D_ may be due to the fact that the study group was composed of healthy, physically active and relatively young people. Furthermore, the four-week period of following this model of nutrition could be relatively too short. Nevertheless, a significant increase in the average concentration of triglycerides was noticed in the Mix_D_ group, unlike in the Veg_D_ group, in which no significant increase in this indicator was observed. These results do not coincide with the results of a systematic review by Yokoyama et al. [45] on the relationship between plant-based diets and the blood lipid profile of people using this model of nutrition. Based on 30 observational studies and 19 clinical trials that met the inclusion criteria (*n* = 1484, mean age: 48.6 years), vegetarian diets were associated with lower mean total (−29.2 and −12.5 mg·dL^−1^, *p* < 0.001), LDL (−22.9 and −12.2 mg·dL^−1^, *p* = 0.001) and HDL cholesterols levels (−3.6 and −3.4 mg·dL^−1^, *p* < 0.001) compared to a Mix_D_ intake in observational and clinical studies, respectively. Mean differences in triglycerides concentrations were −6.5 mg·dL^−1^ (*p* = 0.092) in the observational studies and 5.8 mg·dL^−1^ (*p* = 0.090) in the interventional studies. Study results collected in that systematic review showed that plant-based diets were associated with decreased concentrations of total cholesterol, LDL and HDL cholesterol, but not reduced triglycerides [45]. The results of the above-discussed systematic review correlate with the results obtained after the four-week nutritional interventions during HIFT in the Veg_D_ group only in terms of triglycerides levels. A similar systematic review of studies describing the effect of vegetarian diets on the blood lipid profile (total cholesterol, HDL and LDL cholesterol fractions, and triglycerides) was performed by Wang et al. [46], who, after developing 11 studies meeting the inclusion criteria, also drew the same conclusions about the blood lipids changes under the influence of plant diets as Yokoyama et al. [45]. Moreover, a study by Fontana et al. [47] in athletes showed that a low-protein Veg_D_ in an endurance runners group resulted in lower plasma total cholesterol, LDL-C, triglycerides concentrations and the T-CHOL:HDL-C ratio, and a higher plasma HDL-C concentration, than in a Western diet sedentary group.

Moreover, in the current study, no changes in liver activity markers (ALT, AST) were observed between the studied groups. A large cross-sectional investigation [48] showed that participants with higher adherence to a plant-based diet index and a healthy plant-based diet index had a better profile of liver function tests, whilst the reverse was observed for an unhealthy plant-based diet index. There is only one experimental study [49] investigating the effect of a Veg_D_ on liver enzymes activity, but in non-alcoholic fatty liver disease patients. It was shown that following a Veg_D_, the ALT and AST activities statistically significantly decreased. Therefore, it is difficult to extrapolate these observations to healthy and physically active people.

Despite no statistically significant differences in blood glucose (GLU) concentrations between the Veg_D_ and the Mix_D_ group participants, a noticeable decrease of GLU in the Veg_D_ group and an increase of GLU in the Mix_D_ group may be recognised. A systematic review of six studies on the effect of plant-based diets on blood GLU in non-training subjects by Yokoyama et al. [50] also found a non-statistically significant reduction in fasting blood GLU in plant-based subjects compared to trial participants using a Mix_D_. Moreover, a study on athletes by Fontana et al. [47] showed lower levels of plasma fasting GLU in a low-energy, low-protein Veg_D_ in an endurance runners group than in a Western diet sedentary group. Finally, our study did not show any significant changes in urea and creatinine changes between the groups. In this area, a study by Nebl et al. [51] showed that pre-exercise plasma creatinine levels tended to be higher in omnivorous compared to lacto-ovo-vegetarians and especially to vegans, but did not change significantly post-exercise. Lower creatinine concentrations in the Veg_D_ group were presumably due to the creatine-poor or creatine-free Veg_D_. Moreover, there were no changes in nutritional status markers (protein, albumin) between the groups in our study, but we are not able to compare the results due to the lack of previous studies in this area.

The strength of the research was the compliance and daily evaluation of energy and nutrients’ intake using a mobile application, a properly selected and carefully monitored training specificity and diet program, a homogeneous group of participants in terms of age, training experience and skills. Attention is also drawn to the fact that the volunteers were highly involved and there were no drop-outs—all participants meeting the inclusion criteria and enrolled in the study completed the entire protocol. The limitations of this research include: (1) the relatively short four-week period of nutritional interventions, extension of which could reveal more noticeable differences, (2) monitoring of only resting biochemical markers; however, some additional changes could be observed with simultaneous pre- and post-exercise control, (3) the involvement of people with a moderate level of HIFT training, where extrapolation of their results to elite competitors could be unreliable, and (4) the small sample size, although it was sufficient to detect significant differences. For the above-mentioned reasons, we suggest taking these indications into account when interpreting the results and planning further research in this area.

## 5. Conclusions

In summary, the four-week Veg_D_ and Mix_D_ nutritional interventions during high-intensity functional training improved endurance strength in some exercises and the efficiency of completing training units. Furthermore, the Mix_D_ in HIFT training may support the CrossFit-specific performance. However, the trigger in the above-mentioned elements seems to be the training stimulation of the body and the individual’s predisposition to improve the results in a given exercise. Moreover, in healthy and active people, a properly planned vegan diet does not adversely affect the haematological potential, nor does it affect the lipid profile or markers of liver activity, iron metabolism and nutritional status, or concentrations of glucose, urea and creatinine. Ultimately, therefore, it appears that in moderate-trained people, a vegan diet can be an alternative to a mixed diet for short, intense HIFT exercises, but is unlikely to be more beneficial in improving exercise capabilities than a traditional mixed diet.

## Figures and Tables

**Table 1:** Baseline group characteristics.

Indicator	Vegan Diet (Veg_D_) (*n* = 10: 6 F, 4 M)	Customary Mixed Diet (Mix_D_) (*n* = 10: 6 F, 4 M)	*p*-Value
Age (years)	31.0 ± 3.6	30.5 ± 3.0	0.521
Body mass (kg)	71.6 ± 14.5	72.0 ± 16.1	0.961
Body height (cm)	177 ± 9	173 ± 9	0.304
Fat mass (%)	21.8 ± 4.8	22.5 ± 7.7	0.809
Fat-free mass (kg)	55.8 ± 12.0	55.2 ± 11.7	0.917
Total body water content (%)	53.2 ± 3.4	52.2 ± 5.1	0.600
Squat—1RM (kg)	78.0 ± 24.2	79.5 ± 22.0	0.791
Deadlift—1RM (kg)	87.0 ± 32.7	91.3 ± 27.9	0.758
HITF experience (months)	12.1 ± 5.4	12.1 ± 5.1	1.000

Values are expressed as the mean $\left(\bar{X}\right)$ ± standard deviation (SD). F—females, HIFT—high-intensity functional training, M—males, 1RM—one repetition-maximum.

**Table 2:** Nutritional value of customary and interventional diets.

Variable	Group	Investigation Period		*p*-Value
		Before Nutritional Intervention (T_1_)	After Nutritional Intervention (T_2_)	
		$\bar{X}\pm \mathrm{SD}$ (95% CI)	$\bar{X}\pm \mathrm{SD}$ (95% CI)	
Energy (kcal·day^−1^)	Veg_D_	2346 ± 574	2460 ± 438	0.203
		(1935–2757)	(2147–2773)	
	Mix_D_	2419 ± 361	2420 ± 416	0.991
		(2161–2677)	(2123–2717)	
	*p*-value	0.738	0.762	-
Protein (g·day^−1^)	Veg_D_	110.0 ± 21.9	113.7 ± 23.4	0.508
		(94.3–125.6)	(97.0–130.5)	
	Mix_D_	129.1 ± 14.9	130.1 ± 23.7	0.870
		(118.5–139.7)	(113.1–147.0)	
	*p*-value	**0.034**	0.070	-
Fat (g·day^−1^)	Veg_D_	59.4 ± 9.7	62.3 ± 21.2	0.646
		(52.5–66.3)	(47.1–77.4)	
	Mix_D_	98.9 ± 8.5	98.9 ± 12.7	0.973
		(92.9–105.0)	(89.8–108.0)	
	*p*-value	**<0.001**	**0.002**	-
Carbohydrates (g·day^−1^)	Veg_D_	375.2 ± 120.4	395.3 ± 63.1	0.508
		(289.1–461.3)	(350.1–440.4)	
	Mix_D_	299.7 ± 48.1	297.7 ± 53.9	0.868
		(265.3–334.1)	(259.1–336.2)	
	*p*-value	0.082	**0.010**	-
Dietary fiber (g·day^−1^)	Veg_D_	44.9 ± 9.5	59.7 ± 8.7	**0.007**
		(38.1–51.7)	(53.5–65.9)	
	Mix_D_	39.7 ± 6.3	39.3 ± 5.8	0.575
		(35.2–44.2)	(35.2–43.4)	
	*p*-value	0.104	**<0.001**	-
Water/drinks (ml·day^−1^)	Veg_D_	2704 ± 555	3000 ± 0	0.126
		(2307–3101)	-	
	Mix_D_	2198 ± 525	3000 ± 0	**<0.001**
		(1823–2574)	-	
	*p*-value	0.051	-	-

Values are expressed as means $(\bar{X})\pm \mathrm{SD}$ and 95% CI. Veg_D_—Vegan Diet, Mix_D_—Mixed Diet.

**Table 3:** Summary of the results obtained in the HIFT-specific performance (in the modified ‘Fight Gone Bad’ test) before and after the 4-week nutritional intervention in the Veg_D_ and the Mix_D_ groups.

Variable	Group	Investigation Period		*p*-Value
		Before Nutritional Intervention (T_1_)	After Nutritional Intervention (T_2_)	
		$\bar{X}\pm \mathrm{SD}$ (95% CI)	$\bar{X}\pm \mathrm{SD}$ (95% CI)	
Modified FGB Test				
Wall Ball (reps)	Veg_D_	21.4 ± 6.6	25.8 ± 7.9	**0.041**
		(16.7–26.1)	(20.2–31.4)	
	Mix_D_	22.4 ± 6.8	26.6 ± 3.9	**0.015**
		(17.5–27.3)	(23.8–29.4)	
	*p*-value	0.743	0.776	-
Sumo Deadlift High Pull (reps)	Veg_D_	16.0 ± 8.3	15.9 ± 6.1	0.726
		(10.1–21.9)	(11.5–20.3)	
	Mix_D_	17.2 ± 7.8	15.5 ± 7.1	0.080
		(11.6–22.8)	(10.5–20.5)	
	*p*-value	0.406	0.821	-
Box Jump (reps)	Veg_D_	22.3 ± 7.8	23.0 ± 5.9	0.650
		(16.7–27.9)	(18.8–27.2)	
	Mix_D_	22.4 ± 5.7	23.5 ± 5.5	0.154
		(18.3–26.5)	(19.5–27.5)	
	*p*-value	0.406	0.847	-
Push Press (reps)	Veg_D_	16.7 ± 7.3	14.4 ± 5.9	0.084
		(11.5–21.9)	(10.1–18.7)	
	Mix_D_	16.6 ± 4.8	15.9 ± 5.7	0.550
		(13.2–20.0)	(11.8–20.0)	
	*p*-value	0.971	0.571	-
Rowing (kcal)	Veg_D_	15.5 ± 5.4	16.6 ± 5.9	0.124
		(11.7–19.3)	(12.4–20.8)	
	Mix_D_	14.9 ± 3.3	16.4 ± 3.6	**0.050**
		(12.5–17.3)	(13.8–19.0)	
	*p*-value	0.734	1.000	-
Total FGB_Mod_ completion time (s)	Veg_D_	394.9 ± 14.6	387.8 ± 27.8	0.878
		(384.5–405.3)	(367.9–407.7)	
	Mix_D_	394.3 ± 10.4	393.0 ± 20.2	0.837
		(386.9–401.7)	(378.5–407.5)	
	*p*-value	0.917	0.850	-

Values are expressed as means ($\bar{X}$) ± SD and 95% CI. Veg_D_—Vegan Diet, Mix_D_—Mixed Diet.

**Table 4:** Summary of the level of blood haematological parameters before and after the 4-week nutritional intervention in the Veg_D_ and the Mix_D_ groups.

Variable	Group	Investigation Period		*p*-Value
		Before Nutritional Intervention (T_1_)	After Nutritional Intervention (T_2_)	
		$\bar{X}\pm \mathrm{SD}$ (95% CI)	$\bar{X}\pm \mathrm{SD}$ (95% CI)	
White blood cells (10^9^·L^−1^)	Veg_D_	8.4 ± 2.3	8.2 ± 2.0	0.721
		(6.7–10.0)	(6.8–9.7)	
	Mix_D_	9.5 ± 2.0	8.7 ± 2.3	0.309
		(8.0–10.9)	(7.1–10.4)	
	*p*-value	0.162	0.626	-
Lymphocytes (10^9^·L^−1^)	Veg_D_	3.2 ± 0.8	3.5 ± 0.9	0.188
		(2.7–3.8)	(2.9–4.1)	
	Mix_D_	3.3 ± 1.1	3.1 ± 0.7	0.726
		(2.5–4.1)	(2.6–3.7)	
	*p*-value	0.887	0.324	-
Monocytes (10^9^·L^−1^)	Veg_D_	1.8 ± 3.2	1.8 ± 3.5	0.878
		(−0.5–4.0)	(−0.7–4.3)	
	Mix_D_	0.8 ± 0.2	0.7 ± 0.2	0.392
		(0.6–0.9)	(0.6–0.9)	
	*p*-value	0.970	0.791	-
Granulocytes (10^9^·L^−1^)	Veg_D_	4.4 ± 1.7	4.0 ± 1.3	0.169
		(3.2–5.6)	(3.1–5.0)	
	Mix_D_	5.4 ± 1.1	4.9 ± 1.8	0.236
		(4.6–6.2)	(3.6–6.1)	
	*p*-value	0.104	0.252	-
Red blood cells (10^12^·L^−1^)	Veg_D_	5.75 ± 0.17	5.74 ± 0.14	0.761
		(5.63–5.86)	(5.64–5.85)	
	Mix_D_	5.60 ± 0.21	5.58 ± 0.20	0.558
		(5.45–5.75)	(5.43–5.72)	
	*p*-value	0.092	**0.047**	-
Haemoglobin (mmol·L^−1^)	Veg_D_	9.88 ± 0.16	9.86 ± 0.24	0.875
		(9.77–9.99)	(9.69–10.03)	
	Mix_D_	9.91 ± 0.18	10.00 ± 0.41	0.556
		(9.79–10.04)	(9.70–10.29)	
	*p*-value	0.675	0.390	-
Platelets (10^9^·L^−1^)	Veg_D_	223 ± 61	194 ± 58	0.240
		(180–266)	(153–235)	
	Mix_D_	227 ± 72	193 ± 100	0.119
		(175–278)	(122–265)	
	*p*-value	0.901	0.982	-

Values are expressed as means ($\bar{X}$) ± SD and 95% CI. Veg_D_—Vegan Diet, Mix_D_—Mixed Diet.

**Table 5:** Summary of the concentrations of lipid profile markers before and after the 4-week nutritional intervention in the Veg_D_ and the Mix_D_ groups.

Variable	Group	Investigation Period		*p*-Value
		Before Nutritional Interventional (T_1_)	After Nutritional Interventional (T_2_)	
		$\bar{X}\pm \mathrm{SD}$ (95% CI)	$\bar{X}\pm \mathrm{SD}$ (95% CI)	
Total cholesterol (mg·dL^−1^)	Veg_D_	220.5 ± 34.6	217.1 ± 47.1	0.714
		(195.7–245.2)	(183.5–250.8)	
	Mix_D_	214.5 ± 34.1	214.9 ± 34.8	0.942
		(190.1–238.9)	(190.0–239.8)	
	*p*-value	0.701	0.906	-
High-density lipoprotein cholesterol (mg·dL^−1^)	Veg_D_	72.6 ± 18.0	68.9 ± 21.2	0.172
		(59.7–85.5)	(53.7–84.0)	
	Mix_D_	73.7 ± 24.8	71.5 ± 23.5	0.443
		(56.0–91.4)	(54.7–88.4)	
	*p*-value	0.909	0.793	-
Low-density lipoprotein cholesterol (mg·dL^−1^)	Veg_D_	131.2 ± 34.6	125.1 ± 31.4	0.337
		(106.5–156.0)	(102.6–147.6)	
	Mix_D_	121.5 ± 31.3	113.1 ± 28.3	0.213
		(99.1–143.8)	(92.8–133.3)	
	*p*-value	0.516	0.381	-
Triglycerides (mg·dL^−1^)	Veg_D_	124.8 ± 37.2	143.9 ± 54.9	0.284
		(98.2–151.4)	(104.7–183.2)	
	Mix_D_	110.9 ± 71.6	180.9 ± 113.6	**0.005**
		(59.6–162.1)	(99.6–262.1)	
	*p*-value	0.592	0.791	-

Values are expressed as means ($\bar{X}$) ± SD and 95% CI. Veg_D_—Vegan Diet, Mix_D_—Mixed Diet.

**Table 6:** Summary of the concentrations of other selected biochemical markers before and after the 4-week nutritional intervention in the Veg_D_ and the Mix_D_ groups.

Variable	Group	Investigation Period		*p*-Value
		Before Nutritional Interventional (T_1_)	After Nutritional Interventional (T_2_)	
		$\bar{X}\pm \mathrm{SD}$ (95% CI)	$\bar{X}\pm \mathrm{SD}$ (95% CI)	
Iron (µg·dL^−1^)	Veg_D_	104.6 ± 81.6	108.4 ± 48.1	0.900
		(46.2–162.9)	(74.0–142.9)	
	Mix_D_	115.7 ± 55.5	125.6 ± 62.1	0.672
		(76.1–155.4)	(81.2–170.0)	
	*p*-value	0.724	0.498	-
Transferrin (g·L^−1^)	Veg_D_	3.06 ± 0.64	3.17 ± 0.62	0.213
		(2.60–3.52)	(2.73–3.61)	
	Mix_D_	3.02 ± 0.54	2.86 ± 0.40	0.139
		(2.63–3.40)	(2.58–3.15)	
	*p*-value	0.850	0.273	-
Ferritin (ng·mL^−1^)	Veg_D_	60.4 ± 46.1	56.2 ± 36.7	0.799
		(27.4–93.4)	(30.0–82.5)	
	Mix_D_	84.6 ± 85.2	81.3 ± 74.6	0.959
		(23.7–145.6)	(27.9–134.7)	
	*p*-value	0.970	0.354	-
Protein (g·dL^−1^)	Veg_D_	8.3 ± 0.6	8.7 ± 0.6	0.126
		(7.9–8.8)	(8.3–9.1)	
	Mix_D_	8.7 ± 0.9	9.1 ± 0.6	0.386
		(8.1–9.4)	(8.6–9.5)	
	*p*-value	0.121	0.155	-
Albumin (g·dL^−1^)	Veg_D_	5.4 ± 0.3	5.4 ± 0.2	0.448
		(5.2–5.6)	(5.3–5.6)	
	Mix_D_	5.6 ± 0.6	5.6 ± 0.4	0.778
		(5.2–6.0)	(5.3–5.9)	
	*p*-value	0.264	0.333	-
Alanine aminotransferase (U·L^−1^)	Veg_D_	28.3 ± 9.8	31.0 ± 12.5	0.277
		(21.3–35.3)	(22.1–39.9)	
	Mix_D_	46.7 ± 36.6	50.3 ± 35.5	0.575
		(20.5–72.9)	(24.9–75.7)	
	*p*-value	0.385	0.385	-
Aspartate aminotransferase (U·L^−1^)	Veg_D_	41.7 ± 23.0	43.8 ± 24.0	0.646
		(25.3–58.2)	(26.6–61.0)	
	Mix_D_	49.2 ± 20.0	76.0 ± 91.2	0.721
		(35.0–63.5)	(10.8–141.2)	
	*p*-value	0.307	0.385	-
Glucose (mg·dL^−1^)	Veg_D_	119.9 ± 33.4	103.4 ± 29.4	0.241
		(95.9–143.8)	(82.4–124.5)	
	Mix_D_	106.7 ± 20.7	118.3 ± 26.6	0.254
		(91.9–121.5)	(99.3–137.3)	
	*p*-value	0.303	0.140	-
Urea (mmol·L^−1^)	Veg_D_	7.1 ± 2.3	6.8 ± 2.3	0.627
		(5.5–8.8)	(5.2–8.5)	
	Mix_D_	6.4 ± 1.5	6.7 ± 2.1	0.657
		(5.3–7.5)	(5.1–8.2)	
	*p*-value	0.426	0.866	-
Creatinine (µmol·L^−1^)	Veg_D_	75.1 ± 15.2	78.7 ± 13.5	0.365
		(64.2–86.0)	(69.1–88.3)	
	Mix_D_	80.5 ± 18.6	75.2 ± 26.6	0.386
		(67.2–93.8)	(56.1–94.2)	
	*p*-value	0.521	0.714	-

Values are expressed as means ($\bar{X}$) ± SD and 95% CI. Veg_D_—Vegan Diet, Mix_D_—Mixed Diet.

## Data Availability

The datasets used and/or analysed during the current study are available from the corresponding author upon reasonable request.

## Supplementary Materials

The following supporting information can be downloaded at: https://www.mdpi.com/article/10.3390/nu14040894/s1, Table S1: Minerals and vitamins’ intake in the customary and interventional diets; Table S2: HIFT training protocol; Table S3: The results of the HIFT training units during the 4-week nutritional interventions period.
