# Supplementary material for: Effect of a Four-Week Vegan Diet on Performance, Training Efficiency and Blood Biochemical Indices in CrossFit-Trained Participants
Source: Nutrients. 2022 Feb 20;14(4):894. doi: 10.3390/nu14040894 (PMC8878731; doi:10.3390/nu14040894)
Supplement: Supplementary file 1 [file nutrients-14-00894-s001.zip › Table S1.pdf]

Table S1. Minerals and vitamins intake in the customary and interventional diets

Variable	Group	Investigation period		p-value
		Before nutritional interventional (T_1)	After nutritional interventional (T_2)	
		$\bar{X}$ ± SD (95% CI)	$\bar{X}$ ± SD (95% CI)	
Sodium ($mg \cdot day^{-1}$)	Veg_D	1983 ± 551 (1589 - 2378)	1628 ± 117 (1545 - 1711)	0.074
	Mix_D	1865 ± 534 (1483 - 2246)	1496 ± 73 (1443 - 1548)	0.093
	p-value	0.345	**0.011**	-
Potassium ($mg \cdot day^{-1}$)	Veg_D	3387 ± 584 (2969 - 3805)	5079 ± 865 (4461 - 5698)	**0.007**
	Mix_D	3592 ± 611 (3154 - 4029)	5490 ± 791 (4924 - 6056)	**<0.001**
	p-value	0.453	0.257	-
Calcium ($mg \cdot day^{-1}$)	Veg_D	998 ± 281 (797 - 1199)	1669 ± 243 (1495 - 1843)	**0.001**
	Mix_D	864 ± 243 (690 - 1038)	1109 ± 139 (1010 - 1208)	**0.005**
	p-value	0.269	**<0.001**	-
Phosphorus ($mg \cdot day^{-1}$)	Veg_D	2264 ± 475 (1924 - 2604)	2408 ± 402 (2121 - 2695)	0.074
	Mix_D	2424 ± 336 (2184 - 2665)	2422 ± 410 (2128 - 2716)	0.980
	p-value	0.396	1.000	-
Magnesium ($mg \cdot day^{-1}$)	Veg_D	654 ± 138 (555 - 753)	691 ± 125 (601 - 780)	0.169
	Mix_D	735 ± 117 (651 - 818)	715 ± 120 (629 - 800)	0.488
	p-value	0.175	0.257	-
Iron ($mg \cdot day^{-1}$)	Veg_D	15.8 ± 5.9 (11.6 - 20.0)	27.1 ± 3.9 (24.3 - 29.9)	**0.005**
	Mix_D	17.4 ± 4.7 (14.0 - 20.8)	19.3 ± 3.2 (17.0 - 21.6)	0.176
	p-value	0.525	**0.001**	-
Zinc ($mg \cdot day^{-1}$)	Veg_D	15.30 ± 3.88 (12.52 - 18.07)	16.80 ± 2.59 (14.95 - 18.65)	**0.028**
	Mix_D	15.82 ± 2.65 (13.93 - 17.71)	15.83 ± 2.84 (13.80 - 17.85)	0.959
	p-value	0.791	0.326	-
Copper ($mg \cdot day^{-1}$)	Veg_D	3.54 ± 1.02 (2.81 - 4.27)	3.88 ± 0.51 (3.52 ± 4.24)	0.139
	Mix_D	3.24 ± 0.54 (2.85 - 3.63)	3.15 ± 0.49 (2.80 - 3.50)	0.473
	p-value	0.414	**0.013**	-
Manganese ($mg \cdot day^{-1}$)	Veg_D	88.06 ± 249.30 (-90.28 - 266.40)	246.15 ± 380.48 (-26.04 - 518.33)	0.093

	Mix_D	8.25 ± 1.31 (7.31 - 9.18)	8.08 ± 1.27 (7.17 - 8.99)	0.575
	p-value	0.473	**0.001**	-
Vitamin A (μg·day^{-1})	Veg_D	1775 ± 560 (1374 - 2176)	1938 ± 265 (1749 - 2128)	0.799
	Mix_D	2071 ± 25 (2054 - 2089)	2311 ± 217 (2156 - 2466)	**0.005**
	p-value	0.112	**0.034**	-
Vitamin E (mg·day^{-1})	Veg_D	11.46 ± 5.57 (7.48 - 15.44)	10.74 ± 8.23 (4.85 - 16.63)	0.285
	Mix_D	30.83 ± 2.72 (28.88 - 32.77)	29.53 ± 2.64 (27.64 - 31.42)	**0.026**
	p-value	**<0.001**	**0.002**	-
Thiamine (mg·day^{-1})	Veg_D	2.603 ± 0.453 (2.279 - 2.927)	3.068 ± 0.568 (2.662 - 3.474)	**0.013**
	Mix_D	2.881 ± 0.468 (2.546 - 3.216)	3.254 ± 0.568 (2.848 - 3.660)	0.145
	p-value	0.089	0.257	-
Riboflavin (mg·day^{-1})	Veg_D	3.461 ± 0.938 (2.790 - 4.132)	5.299 ± 0.887 (4.665 - 5.933)	**<0.001**
	Mix_D	2.463 ± 0.591 (2.040 - 2.886)	2.828 ± 0.439 (2.514 - 3.142)	0.112
	p-value	**0.011**	**<0.001**	-
Niacin (mg·day^{-1})	Veg_D	16.13 ± 2.74 (14.17 - 18.08)	18.79 ± 10.67 (11.15 - 26.42)	0.646
	Mix_D	25.00 ± 7.18 (19.86 - 30.14)	38.89 ± 7.00 (33.89 - 43.90)	**0.009**
	p-value	**0.002**	**0.002**	-
Pyridoxine (mg·day^{-1})	Veg_D	2.32 ± 0.69 (1.83 - 2.81)	2.23 ± 1.30 (1.30 - 3.16)	0.093
	Mix_D	3.09 ± 1.02 (2.36 - 3.82)	4.77 ± 0.75 (4.23 - 5.30)	**0.011**
	p-value	**0.045**	**0.002**	-
Vitamin C (mg·day^{-1})	Veg_D	365.4 ± 55.7 (325.6 - 405.2)	326.0 ± 42.4 (295.7 - 356.4)	**0.022**
	Mix_D	289.7 ± 8.7 (283.4 - 295.9)	289.1 ± 7.5 (283.8 - 294.5)	0.959
	p-value	**0.026**	**0.010**	-

Values are expressed as means ($\bar{X}$) ± SD and 95% CI. Veg_D – Vegan Diet, Mix_D – Mixed Diet.
