# Supplementary material for: Effect of a Four-Week Vegan Diet on Performance, Training Efficiency and Blood Biochemical Indices in CrossFit-Trained Participants
Source: Nutrients. 2022 Feb 20;14(4):894. doi: 10.3390/nu14040894 (PMC8878731; doi:10.3390/nu14040894)
Supplement: Supplementary file 1 [file nutrients-14-00894-s001.zip › Table S2.pdf]

Table S2. HIFT training protocol

TRAINING PHASE (1 week)	TRAINING PHASE (3 weeks)
**Weekly schedule:**<br>**3 trainings with a minimum of 1 day break:**<br>Monday - training A<br>Tuesday - free<br>Wednesday - training B<br>Thursday - free<br>Friday - training A<br>Saturday, Sunday - free<br><br>**Training A**<br>a) Warm-up (approx. 15 min) - 8 min run on a treadmill (speed 8.0 km/h, climb 3.0), then World's Greatest Stretch, 20 squats with own body weight, 10 repetitions of "get up", 10 jumps on the chest and dynamic stretching of large muscle groups<br>b) The proper part (20 min) -<br>- chin-ups (for men) x 5 repetitions/chin-ups with resistance rubber (for women) x 10 repetitions<br>- classic push-ups (for men)/push-ups with knee support (for women) x 10 repetitions<br>- Tabata squat x 15 repetitions<br><br>The purpose of the actual part of the training was to perform as many circuits as possible within 20 minutes. Rest was only possible between exercises. The number of circuits completed by each participant was noted in the individual folder of the Microsoft Excel program.<br><br>**Training B**<br>a) Warm-up (approx. 15 min) - 8 min run on a treadmill (speed 8.0 km/h, climb 3.0), then World's Greatest Stretch, 20 squats with own body weight, 10 repetitions of "get up", 10 jumps on the chest and dynamic stretching of large muscle groups<br>b) The proper part (5 circuits, approx. 30 min) -<br>- "fall, rise" (burpees) x 10 repetitions<br>- chin-ups (for men) x 5 repetitions/chin-ups with resistance rubber (for women) x 10 repetitions<br>- raising knees to the cage while hanging on a stick x 10 repetitions<br>- classic push-ups (for men)/push-ups with knee support (for women) x 10 repetitions<br>- Tabata squat x 15 repetitions<br>- short sprints of approx. 10 m x 5 repetitions	**Weekly schedule:**<br>**3 trainings with a minimum of 1 day break:**<br>Monday - training C<br>Tuesday - free<br>Wednesday - training D<br>Thursday - free<br>Friday - training C<br>Saturday, Sunday - free<br><br>**Training C**<br>a) Warm-up (approx. 15 min) - 8 min run on a treadmill (speed 8.0 km/h, climb 3.0), then World's Greatest Stretch, 20 squats with own body weight, 10 repetitions of "get up", 10 jumps on the chest and dynamic stretching of large muscle groups<br>b) The proper part (20 min) -<br>- barbell squat over the head (30 kg for men) x 8 repetitions / squat with a barbell over the head (15 kg for women) x 10 repetitions<br>- barbell rowing (40 kg for men) x 8 reps/barbell rowing (20 kg for women) x 10 repetitions<br>- bench press (60 kg for men) x 8 repetitions/bench press (25 kg for women) x 10 repetitions<br><br>The purpose of the actual part of the training was to perform as many circuits as possible within 20 minutes. Rest was only possible between exercises.<br><br>**Training D**<br>a) Warm-up (approx. 15 min) - 8 min run on a treadmill (speed 8.0 km/h, climb 3.0), then World's Greatest Stretch, 20 squats with own body weight, 10 repetitions "get up", 10 jumps on the chest and dynamic stretching of large muscle groups<br>b) The proper part (5 circuits, approx. 30 min) -<br>- barbell tear (50 kg for men) x 5 repetitions/barbell tear (30 kg for women) x 5 repetitions<br>- barbell squat on shoulders (50 kg for men) x 5 repetitions/barbell squat on shoulders (30 kg for women) x 5 repetitions<br>- chin-ups (for men) x 5 repetitions/chin-ups with resistance rubber (for women) x 10 repetitions<br>- High Pull (40 kg for men) x 5 repetitions / High Pull (20 kg for women) x 10 repetitions

The purpose of the actual part of the training was to perform 5 circuits in 30 minutes. Rest was only possible between exercises.	- push-ups on the bars (for men) x 5-10 repetitions/push-ups on the bars using a resistance rubber (for women) x 5-10 repetitions.<br><br>The purpose of the actual part of the training was to perform 5 circuits in 30 minutes. Rest was only possible between exercises.
