# Supplementary material for: Effect of a Four-Week Vegan Diet on Performance, Training Efficiency and Blood Biochemical Indices in CrossFit-Trained Participants
Source: Nutrients. 2022 Feb 20;14(4):894. doi: 10.3390/nu14040894 (PMC8878731; doi:10.3390/nu14040894)
Supplement: Supplementary file 1 [file nutrients-14-00894-s001.zip › Table S3.pdf]

Table S3. The results of the HIFT training units during the 4-week nutritional interventions period

TRENING	Group	Exercises duration (s) $\bar{X} \pm SD$ (95% CI)	Number of series $\bar{X} \pm SD$ (95% CI)
A1	Veg_D	1181.7 ± 11.5 (1173.5 - 1189.9)	8.8 ± 1.4 (7.8 - 9.8)
	Mix_D	1173.6 ± 35.1 (1148.5 - 1198.7)	8.3 ± 1.8 (7.0 - 9.6)
	p-value	0.850	0.492
A2	Veg_D	1172 ± 19.6 (1158.0 - 1186.0)	9.0 ± 1.4 (8.0 - 10.0)
	Mix_D	1175.2 ± 15.0 (1164.5 - 1185.9)	8.3 ± 1.2 (7.5 - 9.1)
	p-value	0.687	0.364
Veg_D (A1 vs. A2)	*p*-value	0.171	0.361
Mix_D (A1 vs. A2)		0.333	1.000
B	Veg_D	1361.1 ± 249.9 (1182.3 - 1539.9)	-
	Mix_D	1392.4 ± 283.2 (1189.8 - 1595.0)	-
	p-value	0.821	-
C1	Veg_D	1167.7 ± 36.5 (1141.6 - 1193.8)	8.9 ± 1.0 (8.2 - 9.6)
	Mix_D	1176.6 ± 26.1 (1156.5 - 1196.6)	8.6 ± 0.9 (7.9 - 9.2)
	p-value	0.775	0.438
C2	Veg_D	1158.1 ± 36.3 (1132.2 - 1184.0)	9.0 ± 1.1 (8.2 - 9.8)
	Mix_D	1152.2 ± 42.4 (1121.8 - 1182.6)	8.8 ± 0.9 (8.1 - 9.5)
	p-value	0.742	0.623
C3	Veg_D	1138.4 ± 22.9 (1122.0 - 1154.8)	9.4 ± 0.8 (8.8 - 10.0)
	Mix_D	1169.0 ± 26.0 (1150.4 - 1187.6)	9.3 ± 0.8 (8.7 - 9.9)
	p-value	**0.012**	0.791
C4	Veg_D	1160.8 ± 25.3 (1139.6 - 1181.9)	9.6 ± 0.7 (9.0 - 10.2)
	Mix_D	1154.2 ± 35.5 (1128.8 - 1179.6)	9.5 ± 0.8 (8.9 - 10.1)
	p-value	0.666	0.859
C5	Veg_D	1155.0 ± 21.1 (1139.9 - 1170.1)	10.1 ± 0.9 (9.5 - 10.7)
	Mix_D	1151.4 ± 30.8 (1129.4 - 1173.4)	9.6 ± 1.2 (8.8 - 10.4)
	p-value	0.764	0.257
C6	Veg_D	1159.8 ± 26.6 (1140.8 - 1178.8)	10.0 ± 0.7 (9.5 - 10.5)

	Mix_D	1167.6 ± 20.6 (1152.9 - 1182.3)	10.1 ± 0.9 (9.5 - 10.7)
	p-value	0.473	0.970
Veg_D (C1-C6)	*p*-value	0.369	**<0.001**[a]
Mix_D (C1-C6)		0.340	**<0.001**[b]
D1	Veg_D	1313.2 ± 178.0 (1176.4 - 1450.0)	-
	Mix_D	1289.0 ± 216.5 (1134.1 - 1443.9)	-
	p-value	0.795	-
D2	Veg_D	1197.9 ± 222.7 (1038.6 - 1357.2)	-
	Mix_D	1253.2 ± 248.1 (1075.7 - 1430.7)	-
	p-value	0.606	-
D3	Veg_D	1107.1 ± 215.3 (953.1 - 1261.1)	-
	Mix_D	1148.7 ± 246.6 (972.3 - 1325.1)	-
	p-value	0.693	-
Veg_D (D1-D3)	*p*-value	**<0.001**[c]	-
Mix_D (D1-D3)		**0.002**[d]	-

Values are expressed as means ($\overline{X}$) ± SD and 95% CI. Veg_D – Vegan Diet; Mix_D – Mixed Diet; [a] C1 value is lower than C5 and C6, C2 is lower than C6; [b] C1 is lower than C6, C2 is lower than C6; [c] D1 is higher than D2 and D3, D2 is higher than D3; [d] D1 is higher than D3, D2 is higher than D3.
